# Dry-Sand Layer Regulates Soil Moisture Stratification and Plant Community Assembly in the Desert–Oasis Ecotone

**DOI:** 10.3390/biology15211943

**Published:** 2026-10-27

**Authors:** Yanfang Lu, Jie Wang, Jian Ma, Chunxi Zhang, Na Li, Xing Li, Junping Mao, Weiling Zhen

**Affiliations:** 1 Zhangye Academy of Forestry Sciences, Zhangye 734000, China; 2 Research Base for Desert Plants, Zhangye 734000, China; 3 Academy of Animal Science and Veterinary Medicine, Qinghai University, Xining 810016, China

**Keywords:** dry-sand layer, soil moisture stratification, plant community assembly, Heihe River Basin

## Abstract

The desert–oasis ecotone is ecologically fragile, yet the profile-scale role of the Dry-Sand Layer (DSL) in regulating soil moisture stratification and plant community assembly remains poorly understood. We systematically investigated six representative sites across the middle Heihe River Basin (with soil types transitioning from silt loam to aeolian sandy soils), combining multi-depth soil hydro-physical profiling (0–120 cm) with comprehensive vegetation sampling (across 90 nested quadrats recording 37 vascular plant species). A distinct DSL commonly formed at 60–80 cm depth across sandy sites, characterized by a bulk density of 1.39 g. cm^−3^ and a distinct moisture trough (typically < 2.0%), decoupling shallow root zones from subsoil moisture pools (1.10% to 5.09%). This edaphic boundary filtered out deep-rooted species, restricting vegetation to shallow-rooted xerophytic shrubs and grasses (e.g., *Reaumuria soongarica (Pall.) Maxim.*, *Aristida adscensionis* L.) exhibiting high interspecific niche overlap (>0.86) and clustered spatial patterns (*C* > 1). Structural equation modeling indicated that subsoil moisture regulated by the DSL directly drives plant community diversity (path coefficient *β* = +0.54) and coverage (*β* = +0.61). Overall, the DSL serves as an essential eco-hydrological buffer balancing shallow soil moisture retention and subsoil conservation, indicating that ecological restoration in hyper-arid ecotones should prioritize DSL preservation and native shallow-rooted vegetation.

## Simple Summary

In arid regions, extreme water scarcity causes a dry-sand layer to form beneath the soil surface, but its exact role in shaping plant life remains poorly understood. To address this, we investigated six sites across the desert-oasis transition zone in northwestern China to examine how this dry soil layer affects soil moisture and plant communities. We found that this layer acts as a natural gatekeeper: it traps rainfall near the surface for shallow-rooted plants while blocking moisture from evaporating from deeper soil layers. This water-saving barrier prevents large trees from growing but allows specific drought-resistant shrubs and grasses to thrive in clustered patterns. Our results showed that regional ecological restoration projects should avoid planting high-water-consuming trees. Instead, conservation efforts should protect natural dry-sand layers and rely on native, shallow-rooted plants to maintain groundwater levels and ecosystem health in dry regions.

## 1. Introduction

The desert–oasis ecotone represents an indispensable ecological transition zone in arid and semiarid landscapes, playing a pivotal role in maintaining regional energy flux, biogeochemical cycling, and landscape stability [1,2]. Globally, drylands cover over 40% of the terrestrial surface and face escalating climate pressures, making the eco-hydrological dynamics of ecotones a central focus in international dryland ecology [3,4]. In hyper-arid regions such as the Hexi Corridor in northwestern China, water availability constitutes the paramount ecological limiting factor shaping ecosystem structure, primary productivity, and community dynamics [1,5]. The middle reaches of the Heihe River Basin are characterized by an extreme continental desert climate with scarce precipitation (mean annual precipitation ~110 mm), intense solar radiation, and immense evaporative demand (potential evaporation > 2400 mm), resulting in frequent aeolian sand activity, severe soil water deficits, and pronounced ecological fragility [1]. The soil surface layer desiccates rapidly under such hyper-arid atmospheric drivers to form a distinct dry-sand layer (DSL), an edaphic interface commonly developed across the desert–oasis boundary [6,7]. Operationally, the DSL is defined as a distinct subsurface sandy horizon. It features severe desiccation (water content typically < 2.0%, forming a distinct moisture trough between the active root zone and subsoil) and mechanical compaction that disrupts capillary continuity between the subsoil and the atmosphere [8,9]. This measurable and repeatable criterion serves as the objective identification threshold for detecting the DSL across soil profile explorations [10]. The genesis of the DSL is driven by the coupled effects of intense atmospheric evaporative demand, aeolian sediment sorting, coarse soil texture, thermal regime, and restricted capillary rise [9,11]. Episodic rainfall infiltration is rapidly offset by rapid topsoil re-evaporation, causing the desiccated horizon to thicken progressively until a dynamic eco-hydrological equilibrium is established across the soil–atmosphere interface [12,13].

Recent advances utilizing distributed fiber-optic sensing and high-resolution column monitoring have demonstrated that phase changes and vapor transport within this drying zone strictly regulate micro-scale soil moisture profiles [14,15]. The ecological functioning of the DSL has remained a subject of active debate in arid eco-hydrology. Previous studies have suggested that the development of a compacted, highly desiccated sand layer forms a physical barrier that decouples upward and downward moisture fluxes, worsening root-zone conditions, aggravating topsoil drought, and inhibiting seedling emergence, plant establishment, and survival [16]. Conversely, other investigations have demonstrated that once the DSL reaches a threshold thickness, it severs continuous liquid capillary pathways, substantially reducing downward thermal conduction and upward convective vapor flux, thereby safeguarding subsoil moisture pools and maintaining regional hydrological balance [12]. In regions with deep water tables, this moisture-conserving mechanism is widely regarded as an indispensable buffer sustaining perennial xerophytic vegetation and mitigating extreme environmental stress [17].

Although the DSL carries critical ecological importance, previous profile-scale investigations in hyper-arid sandlands have predominantly focused on micro-meteorological evaporation rates, soil thermal-hydraulic column experiments, or physical crust dynamics [12,15], often treating the DSL as a static boundary layer or isolating it from biological components. Consequently, we still lack a mechanistic understanding of how profile-scale DSL moisture stratification and structural compaction interactively regulate plant community assembly, floristic diversity, and synusial architecture, particularly across regional desert–oasis ecotones [18,19]. While broader regional hydrological assessments have quantified oasis water balances or general vegetation distribution [1], these studies cannot resolve the fine-scale eco-hydrological feedback operating across the 0–120 cm soil profile. Specifically, previous studies rarely integrate multi-depth soil hydro-physical properties with nested shrub–herb surveys, spatial point-pattern analysis, and structural equation modeling (SEM) to disentangle how subsoil moisture buffers plant performance while the DSL simultaneously acts as a physical exclusion barrier.

To address these knowledge gaps, this study systematically investigated six representative survey plots across the desert–oasis ecotone of the middle Heihe River Basin in Ganzhou, Linze, and Gaotai counties. Unlike previous works that evaluated soil desiccation or vegetation patterns independently, this study combined multi-depth soil hydro-physical measurements, nested vegetation surveys, spatial point-pattern analysis, redundancy analysis (RDA), and SEM to address four objectives: (1) quantify the vertical stratification of soil bulk density and moisture associated with the DSL across sandy habitats; (2) evaluate alpha diversity gradients, niche breadth, and interspecific overlap between shrub and herbaceous synusiae; (3) characterize spatial aggregation patterns of dominant desert plant populations; and (4) elucidate the eco-hydrological pathways by which soil compaction and DSL dynamics regulate plant community structure. The findings provide a scientific theoretical basis for optimizing vegetation restoration configurations, selecting appropriate sand-stabilizing species, and maintaining hydrological sustainability in arid ecotones.

## 2. Materials and Methods

### 2.1. Study Area Characterization

**Figure 1:**
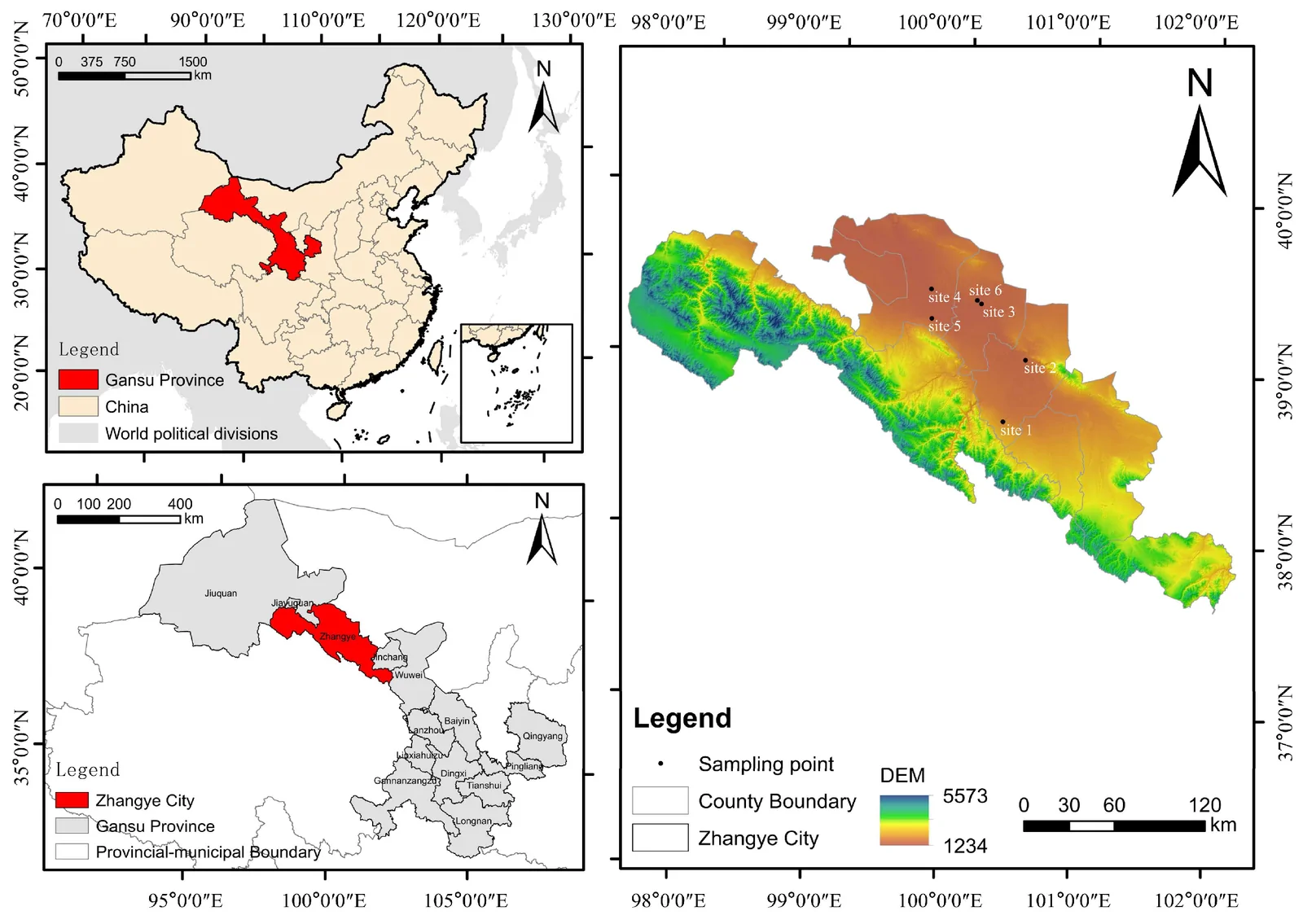
Spatial distribution of six study sites.

This study was conducted in the desert–oasis ecotone situated within the middle reaches of the Heihe River Basin, spanning Ganzhou District (38°42′–39°04′ N, 100°21′–100°31′ E), Linze County (39°22′–39°24′ N, 100°07′–100°09′ E), and Gaotai County (39°17′–39°27′ N, 99°45′–99°46′ E) in Zhangye City, Gansu Province, China (Figure 1). The study area ranges in elevation from 1344.8 to 1848.9 m. This area exhibits an extreme continental desert climate, with a mean annual temperature of 7.8 °C, mean annual precipitation of 110 mm, and intense potential evaporation exceeding 2400 mm. According to the World Reference Base for Soil Resources, the regional soils predominantly transition from Calcisols (in the alluvial silt loam habitats, Site 1) to Arenosols (in the aeolian sandy dune systems, Sites 2–6) [20]. The natural vegetation is sparse and structurally simple, primarily composed of extreme xerophytic and salt-tolerant shrubs (*Reaumuria soongarica* (Pall.) Maxim., *Salsola passerina* Bunge, *Nitraria tangutorum* bobrov, *Sympegma regelii* Bunge) and shallow-rooted companion grasses (*Aristida adscensionis* L., *Carex tristachya* Thunb., *Cleistogenes songorica* (Roshev.) Ohwi).

### 2.2. Nested Sampling Design and Vegetation Community Inventory

To establish a robust comparative framework across the desert–oasis ecotone, a rigorous nested sampling design was implemented to evaluate both vegetation attributes and soil hydro-physical parameters [21]. Field surveys were executed along the hydrological gradient of the middle Heihe River during the peak vegetative growing season (June, August, and October 2025). Six typical long-term survey sites were established across the core ecotone zone: Ganzhou Nantan (Site 1), Ganzhou Tu’erbatan (Site 2), Linze Banqiao (Site 3), Gaotai Heiquan (Site 4), Gaotai Nanhua (Site 5), and Linze Sand Control Farm (Site 6) (Table 1). At each study site, five 50 m × 50 m core survey plots were laid out (totaling 30 macro-plots). Within each 50 m × 50 m plot, three 4 m × 4 m shrub subplots and three nested 1 m × 1 m herbaceous quadrats were systematically established, resulting in a total of 90 shrub and 90 herbaceous sampling units. Within each quadrat, floristic attributes were recorded, including species identity, individual abundance, plant height, crown dimensions, growth form (shrub, semi-shrub, perennial herb, annual/biennial herb), and total synusial coverage. To properly match the hierarchical nested sampling design (sub-quadrats nested within 30 macro-plots across 6 study sites), data collected from individual sub-quadrats (shrub and herbaceous quadrats) were aggregated to the macro-plot scale (N = 30) for soil–vegetation relationships, multivariate ordinations (such as RDA), and SEM. This aggregation and multi-level structuring ensured that the statistical analyses accurately reflected the hierarchical design and preserved robust degrees of freedom across spatial gradients.

### 2.3. Soil Profile Sampling and Hydro-Physical Determinations

Soil profile investigations were conducted across the survey plots in February, April, June, August, October, and December 2025 to capture vertical hydro-physical variations.

New soil profile pits were excavated each bimonthly campaign at undisturbed adjacent locations within the same microtopographic unit, with a 2–3 m buffer from prior pits. This sampling strategy preserves temporal continuity and prevents cumulative local disturbance. Five pits per site were positioned <5 m from the central 50 m × 50 m vegetation macro-plots to guarantee strong geostatistical linkage between soil and vegetation surveys. Profile morphology was characterized during excavation, including horizon boundaries, soil color, compaction interfaces, and root distribution. Adhering to our multi-replicate analytical design, at each survey site, five vertical profile pits were excavated to a depth of 120 cm (totaling 30 profile pits). Undisturbed and homogenized soil samples were collected across seven depth intervals: 0–10, 10–20, 20–40, 40–60, 60–80, 80–100, and 100–120 cm.

Bulk density was determined using standard stainless-steel cutting rings (volume: 100 cm^3^, inner diameter: 50.46 mm, height: 50 mm) in triplicate for each depth. Undisturbed core samples were oven-dried at 105 °C to a constant mass and weighed using an analytical electronic balance (Yoke, Shanghai, China) with an accuracy of 0.001 g. Concurrently, fresh homogenized soil samples were sealed immediately in aluminum containers, transported to the laboratory, weighed for fresh mass, and oven-dried at 105 °C for 24 h to constant dry mass to calculate gravimetric water content (%) in triplicate [22]. Soil particle-size distributions were quantified using the hydrometer method. Pre-treated soil samples (organic matter removed with 10% H_2_O_2_ and carbonates with 0.5 M HCl) were dispersed by adding 50 mL of 0.5 M sodium hexametaphosphate solution (Shanghai Macklin Biochemical Co., Ltd., Shanghai, China) as a chemical dispersant, followed by mechanical high-speed stirring for 10 min. The suspension was transferred to a sedimentation cylinder. Soil particle fractions were classified according to the USDA textural system as sand (0.05–2.0 mm), silt (0.002–0.05 mm), and clay (<0.002 mm). These fractions were determined via hydrometer readings collected at calibrated time intervals, with temperature corrections applied to all measurements (Table S1) [20,23].

### 2.4. Quantitative Ecological Analysis and Diversity Indices

Importance Value (*IV*): The ecological dominance of each plant species within the shrub and herbaceous layers was determined using the importance value index [21]:

*IV* = Relative Density (RD) + Relative Coverage (RC) + Relative Frequency (RF)

where *RD* = (individuals of a species/total individuals) × 100%; *RC* = (coverage of a species/total coverage) × 100%; and *RF* = (quadrat frequency of a species/total species frequencies) × 100%.

Alpha diversity indices: community alpha diversity was evaluated using four standard mathematical metrics: Shannon–Wiener Diversity Index (*H*):

$$H=-\sum_{i=1}^{s}P_{i}\ln P_{i}$$

Simpson Diversity Index (*D*):

$$D=1-\sum_{i=1}^{s}p_{i}^{2}$$

Pielou Evenness Index (*E*):

$$E=\frac{H}{\mathrm{lnS}}$$

Margalef Richness Index (*F*):

$$F=\frac{S-1}{\ln N}$$

where *S* is the total number of recorded species in the quadrat, *N* is the total number of individuals of all species, and *Pi* = *Ni*/*N* represents the proportional abundance of the *i*-th species.

Spatial Point Pattern and Aggregation Indices:

The horizontal distribution pattern of dominant plant populations was determined using classical spatial aggregation metrics [24]:

Dispersion Coefficient (*C*): *C* = *V*/*m*. Here, *m* is the mean population density across quadrats, and *V* is the sample variance. *C* = 1.0 indicates a random distribution, *C* < 1.0 indicates a uniform distribution, and *C* > 1.0 indicates an aggregated (clumped) distribution.

Clumping Index (*I*): $I=\frac{V}{m}-1$. I = 0 denotes random, I < 0 uniform, and I > 0 clumped distribution.

Mean Crowding (M^∗^): $M^{*}=m+(\frac{V}{m}-1)$.

Patchiness Index (PAI): *PAI* = *M*^∗^/*m*. *PAI* > 1.0 indicates significant spatial clustering.

Niche Breadth and Interspecific Overlap:

Shannon–Wiener Niche Breadth (*B_sw_*):

$$B_{\mathrm{sw}}=-\sum_{j=1}^{R}P_{\mathrm{ij}}\ln P_{\mathrm{ij}}$$

where *P_ij_* = *n_ij_*/*N_i_*, representing the proportion of individuals of the *i*-th species occurring in the *j*-th resource state (plot), and R is the total number of surveyed resource states (R = 6).

Pianka Interspecific Niche Overlap Index (*O_ik_*):

$$O_{\mathrm{ik}}\frac{\sum_{j=1}^{R}P_{\mathrm{ij}}P_{\mathrm{kj}}}{\sqrt{\sum_{j=1}^{R}P_{\mathrm{ij}}^{2}}\sum_{j=1}^{R}P_{\mathrm{ij}}^{2}}$$

where *O_ik_* measures the degree of resource utilization overlap between species *i* and species *k* (0 < *O_ik_* < 1).

### 2.5. Multivariate Statistical Modeling and Visualization

All statistical computations and graphic renderings were executed in R version 4.3.2 [25]. Bivariate relationships between soil water content and bulk density were evaluated using ordinary least squares (OLS) linear regression to identify general trends, with potential confounders further addressed in our multivariate models. Differences in diversity metrics among sites and between synusiae were tested using one-way Analysis of Variance (ANOVA) followed by Tukey’s Honest Significant Difference (HSD) post hoc comparison (α = 0.05). Soil hydro-physical variables were temporally matched and averaged using data collected exclusively during the peak vegetation growing season (June, August, and October 2025) to guarantee precise ecological alignment. All community-level multivariate analyses, including RDA and SEM, were synchronized with vegetation surveys. To decipher the multivariate relationships between environmental variables and plant community properties, RDA was conducted using the vegan package in R. Prior to ordination, biological response variables were Hellinger-transformed and environmental matrices were standardized [26]. A SEM was constructed using the lavaan package in R to quantify direct and indirect causal pathways linking edaphic compaction, DSL water filtering, subsoil moisture pools, and community structural diversity. Model fit was evaluated using the chi-square test (χ^2^, *p* > 0.05), Comparative Fit Index (*CFI* > 0.95), and Root Mean Square Error of Approximation (RMSEA < 0.05) [27]. Graphics were rendered using ggplot2, patchwork, and ggrepel packages.

## 3. Results

### 3.1. Habitat Attributes, Soil Textural Features, and Spatial Patterns of Bulk Density and the DSL Across the Study Ecotone

The survey across the desert–oasis ecotone of the middle Heihe River basin encompassed six representative sites spanning Ganzhou, Linze, and Gaotai counties (Table 1). The regional topography sloped northwestward along the river flow, with elevation decreasing from 1848.9 m at Site 1 (Ganzhou Nantan) to 1344.8 m at Site 4 (Gaotai Heiquan). Site 1 was characterized by silt loam with a total vegetation cover of 64.57%, co-dominated by the subshrub *S. passerina* and the perennial sedge *C. tristachya*. In contrast, aeolian sandy soils dominated Sites 2, 3, 4, 5, and 6, where the xerophytic shrub *R. songarica* served as the sole constructive shrub species. Total community coverage ranged between 19.67% (Site 5, Gaotai Nanhua) and 79.01% (Site 6, Linze Sand Control Farm), exhibiting marked structural heterogeneity driven by edaphic texture and localized microrelief.

Soil bulk density across the 0–120 cm profiles exhibited distinct vertical stratification (Table 2). Mean bulk density across all sites peaked in the shallow surface layer (0–10 cm: 1.63 ± 0.15 g.cm^−3^) and the sub-surface horizon (40–60 cm: 1.53 ± 0.12 g.cm^−3^). The DSL was consistently detected at depths of 60–80 cm and exhibited an average bulk density of 1.39 ± 0.06 g.cm^−3^. Across all sandy dune profiles, bulk density decreased progressively with depth beneath the DSL, reaching a profile minimum of 1.29 ± 0.09 g.cm^−3^ at 100–120 cm.

### 3.2. Vertical Soil Moisture Stratification and Water–Compaction Coupling

**Figure 2:**
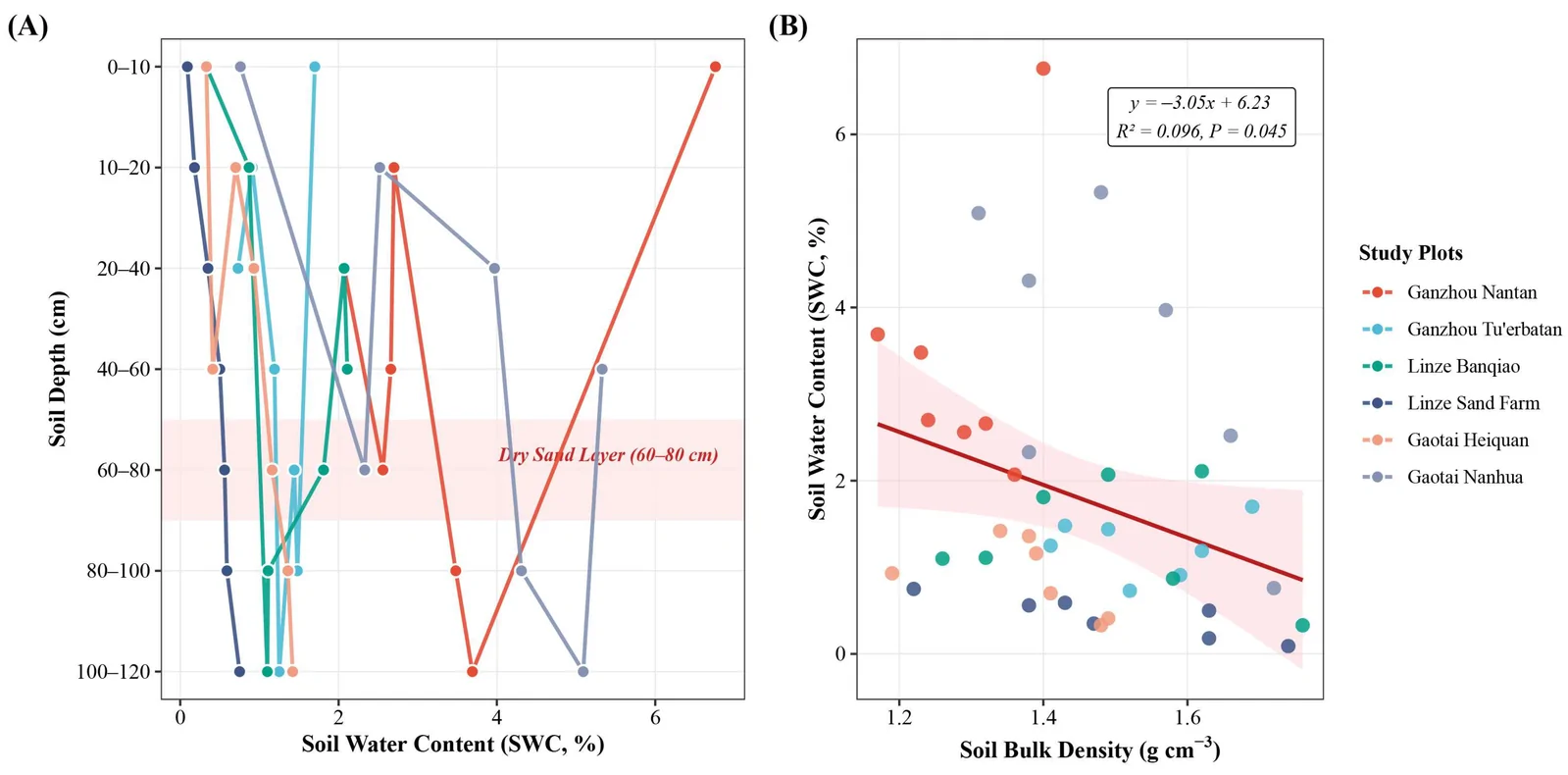
Soil hydro-physical profiles across the 0–120 cm depth and regression relationship in the middle Heihe River ecotone: (**A**) Vertical distribution profiles of soil water content (SWC, %) across six typical survey plots (60–80 cm). (**B**) Linear regression between SWC and soil bulk density across all soil horizons.

Gravimetric soil water content (SWC) ranged from 0.09% to 6.76% across the entire profile, indicating severe water deficits throughout the ecotone (Figure 2A). Across most sandy dune habitats, the vertical distribution of SWC generally follows a characteristic S-shaped increase–decrease–increase profile, yet it exhibited localized variations among individual plots. Notably, sites with distinct surface conditions, such as Gaotai Heiquan with gravelly sand and Linze Sand Farm with high vegetation cover, exhibited weaker or altered moisture fluctuations along the soil profile. In the surface layer (0–20 cm), intense atmospheric evaporation maintained moisture below 2.0% in most sandy habitats. SWC increased to a local peak within the 20–60 cm layer (reaching 5.33% at Site 6). At the 60–80 cm depth, moisture declined abruptly to a profile minimum across all sandy plots. Below 80 cm, soil moisture is replenished and remains stable (ranging from 1.10% to 5.09% in the 100–120 cm horizon). Bivariate regression analysis indicated a statistically significant negative linear relationship between SWC and soil bulk density (Figure 2B), indicating that elevated compaction and reduced macro-porosity directly reduce moisture retention capacity.

### 3.3. Floristic Composition, Importance Values, and Taxonomic Spectrum

A total of 37 vascular plant species representing 31 genera and 12 families were recorded across the 90 surveyed quadrats (Table 3 and Table S2). The community exhibited a typical desert-adapted flora dominated by Poaceae (8 species), Chenopodiaceae (8 species), Asteraceae (6 species), and Fabaceae (3 species). Shrubs and semi-shrubs comprised 27.0% of total species richness (10 species), while perennial and annual/biennial herbs accounted for 73.0% (27 species); arboreal taxa were entirely absent. Among woody shrubs, *R. songarica* had the highest Importance Value (*IV* = 111.17%), followed by *S. passerina* (*IV* = 54.90%), *N. tangutorum* (*IV* = 24.42%), and *S. regelii* (*IV* = 20.11%). In the herbaceous synusia, the annual grass *A. adscensionis* (*IV* = 48.62%) and perennial sedge *C. tristachya* (*IV* = 23.39%) dominated, accompanied by *Chloris virgata* Sw. (*IV* = 18.22%), *Bassia dasyphylla* (Fisch. et Mey.) (*IV* = 13.61%), and *C. songorica* (*IV* = 13.61%).

### 3.4. Alpha Diversity Gradients Between Vegetation Synusiae

**Figure 3:**
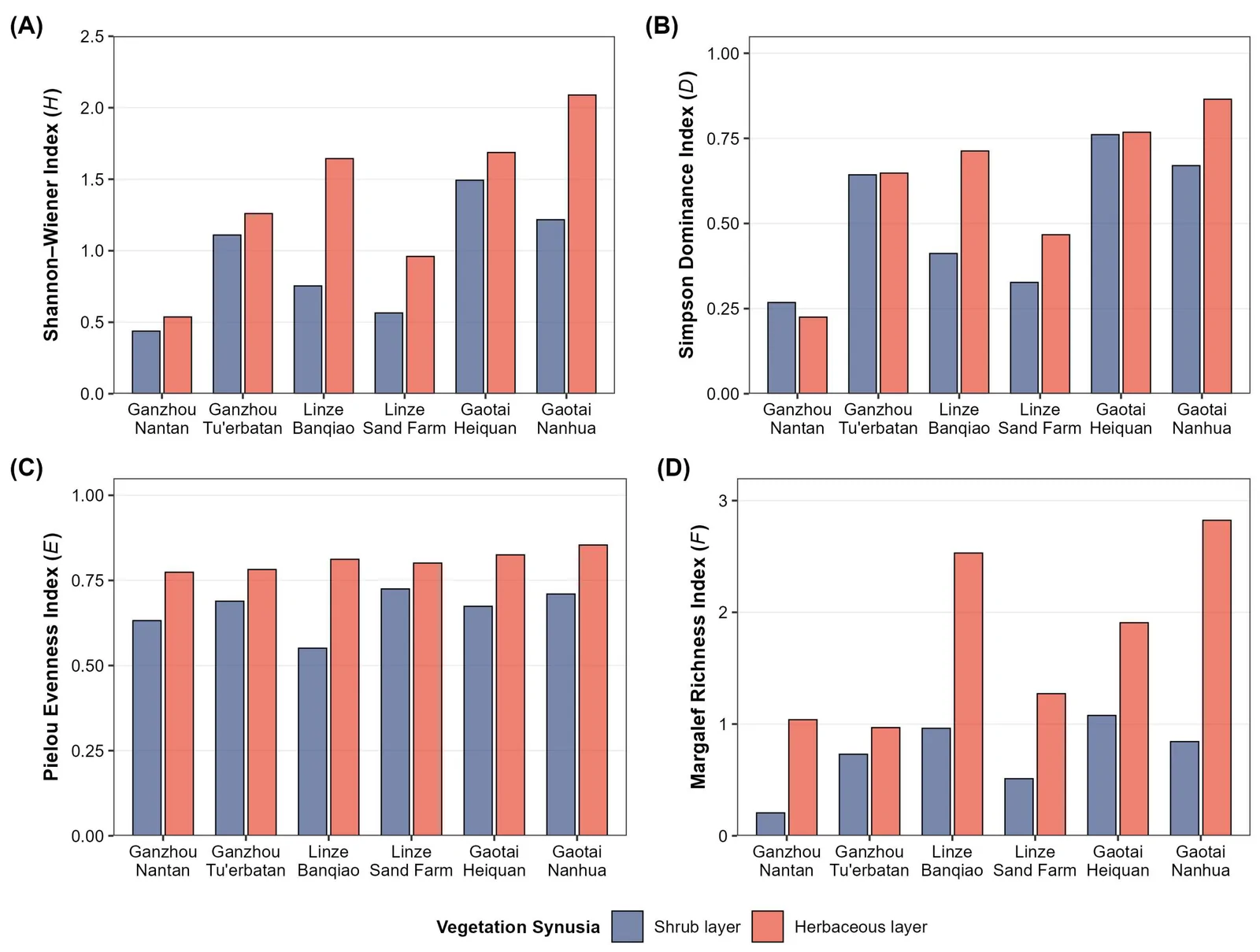
Plant community alpha diversity metrics across shrub and herbaceous layers in the six survey plots: (**A**) Shannon–Wiener diversity index (*H*). (**B**) Simpson diversity index (*D*). (**C**) Pielou evenness index (*E*). (**D**) Margalef richness index (*F*).

The herbaceous layer maintained higher species diversity, structural evenness, and richness compared with the shrub layer across the ecotone (Figure 3A,C,D and Table S3). The Shannon–Wiener diversity index (*H*) in the herbaceous synusia ranged from 0.537 to 2.089, whereas in the shrub synusia it remained lower (*H* = 0.438–1.493) (Figure 3A and Table S3). Similarly, the Margalef richness index (*F*) was substantially elevated in the herbaceous layer (*F* = 0.968–2.824) relative to the shrub layer (*F* = 0.205–1.077), indicating that ephemeral and perennial grasses contribute the vast majority of community species richness (Figure 3D and Table S3). Community dominance characteristics captured by the Simpson index (*D*) further resolved synusial structural divergence across plots (Figure 3B and Table S3). The Simpson index for the herbaceous layer ranged from 0.225 to 0.865, generally exceeding or matching that of the shrub layer (*D* = 0.268–0.761). In sandy habitats with higher species coexistence (Gaotai Nanhua, Gaotai Heiquan, and Linze Banqiao), the herbaceous synusia exhibited elevated Simpson diversity values (*D* = 0.713–0.865), reflecting a more equitable distribution of individuals among co-occurring grass species (such as *A. adscensionis*, *C. songorica*, and *C*. *virgata*). Conversely, in the shrub layer of Ganzhou Nantan (*D* = 0.268), Linze Sand Farm (*D* = 0.327), and Linze Banqiao (*D* = 0.412), the markedly lower Simpson values indicated intense ecological dominance by a single constructive species, primarily *R. soongarica* and *S. passerina*. Spatial heterogeneity in diversity indices closely mirrored the regional topographical and soil textural gradient. Gaotai Nanhua (Site 5) and Gaotai Heiquan (Site 4) exhibited the highest overall community diversity across all metrics (*H* > 1.68, *D* > 0.76, *E* > 0.8, *F* > 1.90 for herbs) (Figure 3A–D). In contrast, Ganzhou Nantan (Site 1) exhibited the lowest alpha diversity across both layers (*H* = 0.438 for shrubs, *H* = 0.537 for herbs; *D* = 0.268 for shrubs, *D* = 0.225 for herbs; *E* = 0.693 for shrubs) (Figure 3A–C). This pattern indicated that while fine-textured silt loam habitats at higher elevations sustain higher total vegetative cover, their synusial structure is constrained by extreme monodominance, whereas sandy ecotones support more evenly distributed, multi-species herbaceous assemblages beneath the DSL boundary.

### 3.5. Niche Breadth and Interspecific Resource Overlap

**Figure 4:**
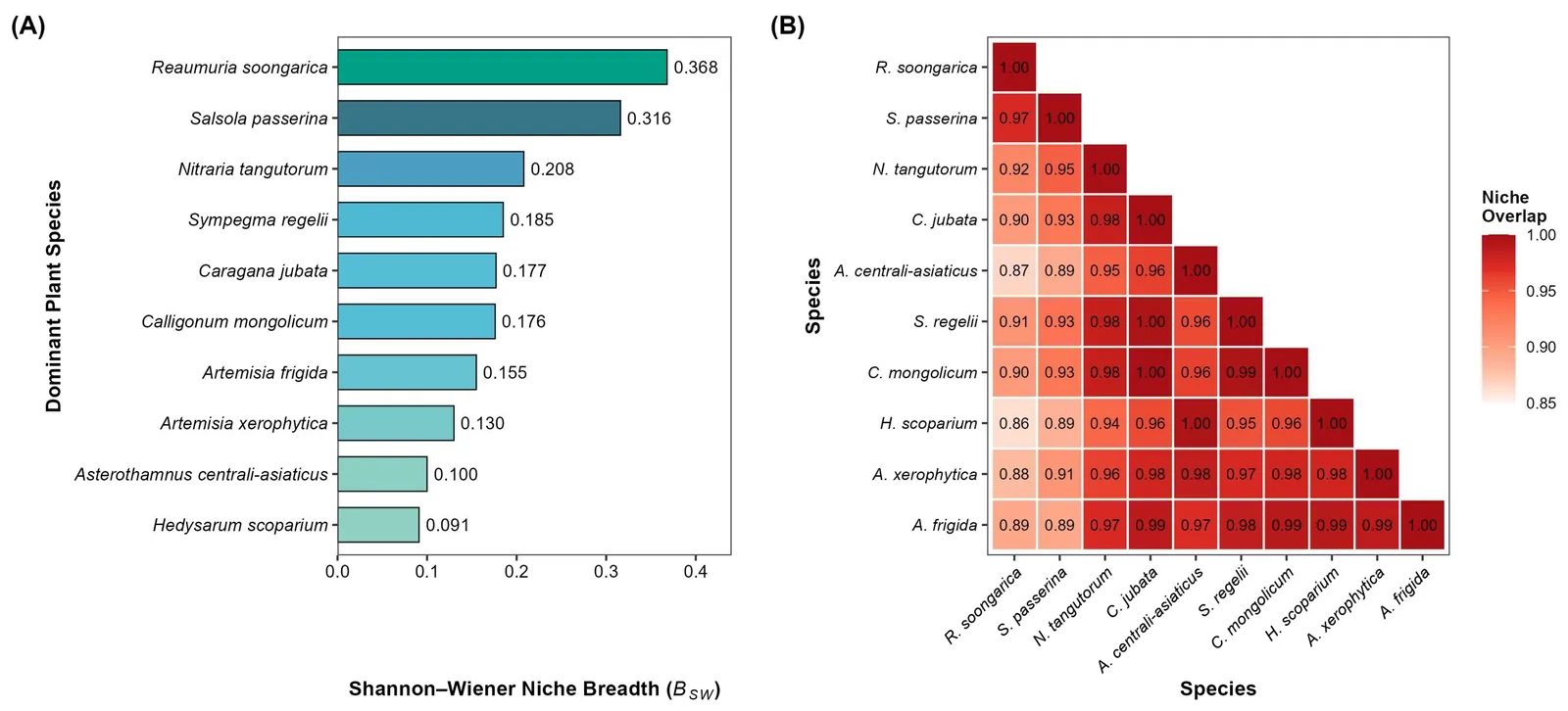
Niche breadth and interspecific niche overlap matrix of dominant plant species: (**A**) Shannon–Wiener niche breadth (*B_SW_*) of dominant xerophytic taxa. (**B**) Symmetrical half-matrix heatmap depicting Pianka’s interspecific niche overlap coefficients.

Shannon–Wiener niche breadth (*B_sw_*) quantified resource exploitation capacities among dominant species (Figure 4A). Constructive xerophytic shrubs occupied the broadest ecological niches: *R. songarica* displayed the highest niche breadth (*B_SW_* = 0.368), followed by *S*. *passerina* (*B_SW_* = 0.316), *N. tangutorum* (*B_sw_* = 0.208), *S. regelii* (*B_SW_* = 0.185), and *C. jubata* (*B_SW_* = 0.177). In contrast, companion shrubs such as Asterothamnus centraliasiaticus (*B_SW_* = 0.100) and *H. scoparium* (*B_SW_* = 0.091) have restricted niche breadths, reflecting narrower micro-habitat specializations. The Pianka niche overlap half-matrix revealed high interspecific overlap coefficients across most dominant species pairs (> 0.86), reflecting a high similarity in their spatial-environmental distribution rather than direct competitive equivalence (Figure 4B). Overlap reached near-unity between *Caragana jubata* and *C. mongolicum* (0.999), *S. regelii* and *C. jubata* (0.996), and *R. songarica* and *S. passerina* (0.974). This indicates convergent resource utilization strategies, with co-occurring species exploiting similar soil moisture pools above the DSL.

### 3.6. Spatial Aggregation Patterns of Desert Plant Populations

**Figure 5:**
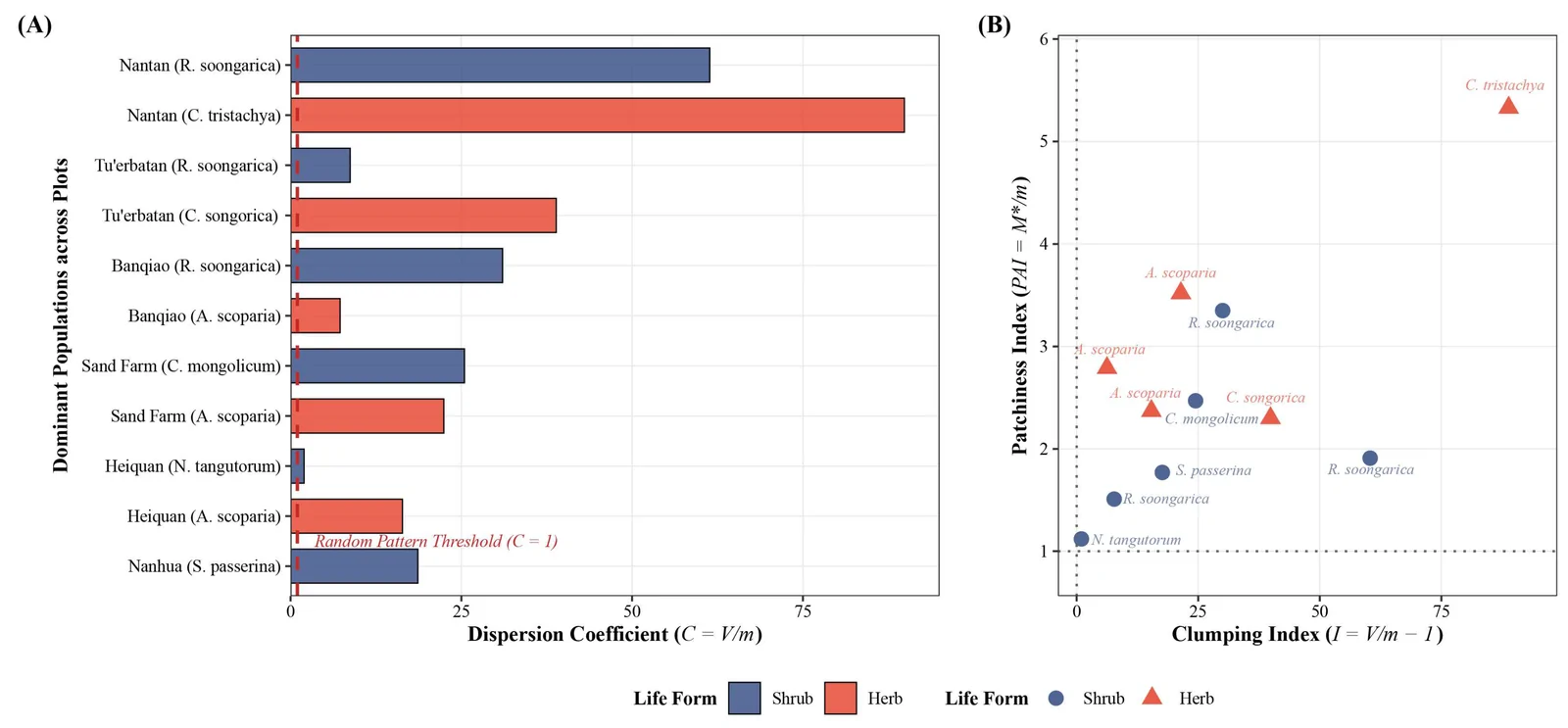
Spatial aggregation metrics of dominant plant populations across survey plots: (**A**) Dispersion coefficient of dominant species. (**B**) Bivariate distribution of clumping index (*I*) versus patchiness index (PAI).

Spatial point-pattern analyses revealed that desert vegetation in the middle Heihe River ecotone conformed to clumped spatial distributions (Figure 5). The dispersion coefficients for all dominant woody shrubs and herbaceous taxa were greater than the critical random threshold of *C* = 1, ranging between 1.98 and 89.85 (Figure 5A). Herbaceous populations displayed markedly higher aggregation intensities than woody shrubs, exemplified by *C. tristachya* in Nantan (*C* = 89.85), *C. songorica* in Tu’erbatan (*C* = 38.89), and *A. scoparia* in Sand Farm (*C* = 22.44). Among shrubs, *R. songarica* exhibited pronounced spatial clustering at Nantan (*C* = 61.36) and Banqiao (C = 31.03). Similarly, the clumping index (*I* = *V*/*m* − 1 > 0) and patchiness index (PAI > 1, with values ranging from 1.11 to 5.32) were elevated across all populations (Figure 5B), indicating that drought stress and localized moisture patches are closely associated with aggregated population architectures.

### 3.7. Multivariate Eco-Hydrological Driving Mechanisms

**Figure 6:**
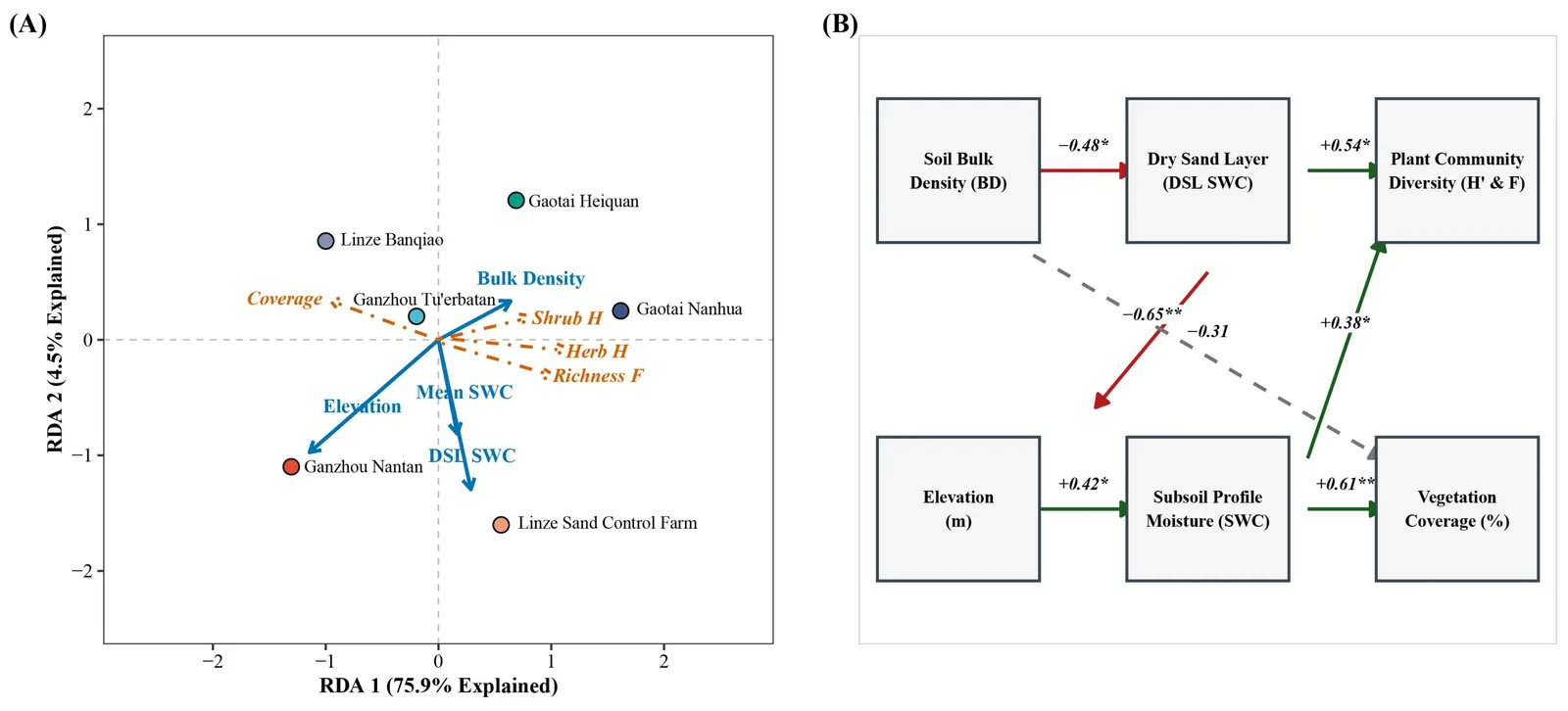
Multivariate driving mechanisms linking soil hydro-physical properties to plant community patterns: (**A**) RDA biplot showing ordination of study plots (where different colored dots represent the six representative survey sites across Ganzhou, Linze, and Gaotai counties, as detailed in Table 1), environmental vectors (blue solid arrows), and vegetation response metrics (orange dash-dotted arrows). (**B**) SEM detailing cascading path coefficients among soil physical structure, DSL dynamics, profile moisture, and plant community attributes (* *p* < 0.05, ** *p* < 0.01).

RDA resolved the multivariate linkages between soil hydro-physical drivers and vegetation community properties (Figure 6A). The first two canonical axes accounted for 80.4% of total variation (RDA1: 75.9%, RDA2: 4.5%). Soil bulk density (BD) and dry-sand layer moisture (DSL SWC) aligned along RDA1, governing the ordination of Gaotai plots (Sites 4 and 5) and correlating positively with herbaceous diversity, shrub diversity, and Margalef richness. Total vegetation cover was oriented along RDA2, associating with elevation and mean profile water content in silt loam habitats (Site 1). SEM evaluated the hypothesized cascading pathways (Figure 6B). Elevated soil bulk density negatively affected DSL moisture retention (path coefficient = −0.48, *p* < 0.05). In turn, DSL desiccation and compaction exerted an inhibitory boundary effect on subsoil water replenishment (−0.65, *p* < 0.01). While shallow-rooted xerophytic shrubs and herbaceous species predominantly extract moisture from the active 0–60 cm rooting zone, the statistical linkage between deep profile moisture reserves and community attributes (+0.54 for alpha diversity, +0.61 for vegetation cover) reflects a critical eco-hydrological buffer mechanism rather than direct root uptake from below the DSL. Based on a sample size of N = 30 macro-plots, the evaluated SEM achieved a robust statistical fit, satisfying all a priori criteria (*χ^2^* = 2.450, *df* = 2, *P* = 0.294, *CFI* = 0.985, RMSEA = 0.038), which indicates the robustness and adequacy of the structural network.

## 4. Discussion

Across sand dune profiles of the middle Heihe River ecotone, the DSL develops at 60–80 cm depth with a defined spatial configuration, forming a key pedogenic and eco-hydrological boundary typical of hyper-arid transition zones [28]. Soil water-holding capacity, the fundamental property determining the efficacy of soil water storage and effective plant moisture availability, is governed by particle packaging, pore geometry, and mechanical compaction [29]. In the middle Heihe River basin, the DSL was predominantly concentrated within the 60–80 cm horizon with an average thickness of 12–20 cm, consistently exhibiting a pronounced moisture trough and a distinct soil bulk density (1.39 ± 0.06 g.cm^−3^) across the surveyed profile. This localized compaction and low water retention capability are tightly linked to the edaphic texture [30]. The DSL consists of > 90% coarse sand particles with low silt content and reduced macro- and capillary porosity, which substantially limit unsaturated hydraulic conductivity relative to the overlying silt loam and underlying sandy loam horizons [31,32]. Under an extreme continental desert regime with low mean annual precipitation (~110 mm) and high potential evaporation (>2400 mm), this desiccated horizon is proposed to play a pivotal dual regulatory role in regional soil water balance. On the one hand, the compacted matrix and high bulk density of the DSL attenuate rapid downward gravitational percolation, preventing limited meteoric recharge from draining into deeper inaccessible zones and thereby retaining ephemeral precipitation within the upper 0–60 cm soil compartment accessible to shallow root networks. On the other hand, the DSL effectively acts as a physical barrier in the capillary network, potentially restricting liquid water transport pathways between the moist subsoil and the atmosphere, reducing downward solar thermal radiation into deeper sand bodies, and suppressing upward evaporative vapor flux [12,33,34]. This maintains subsoil moisture pools beneath 80 cm in a relatively stable state (1.10–5.09%), serving as an essential hydrological buffer that sustains xerophytic vegetation under hyper-arid stress [34,35].

The distinct S-shaped increase–decrease–increase vertical profile of soil moisture observed across the study sites refines traditional models of soil water stratification in arid inland basins (excluding Linze Sand Farm and Gaotai Heiquan). Classical pedological frameworks categorize vertical soil moisture profiles in arid ecosystems into four layers: an evaporation-sensitive layer, a rapidly changing root-absorption layer, a slowly varying transitional layer, and a stable subsoil layer. These layers are governed by atmospheric forcing, vegetation uptake, and capillary recharge [36]. In the middle reaches of the Heihe River, the 0–10 cm horizon forms the evaporation-sensitive zone characterized by low soil water content. The 20–40 cm layer is the rapidly changing zone exploited by plant root networks, where soil moisture rises with increasing depth. The 40–60 cm layer represents a slowly varying zone with a localized moisture peak, and the 60–120 cm horizon is the deep zone influenced by subsoil water reserves. However, the physical interposition of the DSL at 60–80 cm radically shifts the boundary dynamics of this stratification. The 0–120 cm sand body in these dunes receives negligible direct capillary ascent from the deep regional groundwater table. Thus, precipitation pulses supply only the surface sand body. The DSL forms a pronounced hydrological inflection point where moisture drops precipitously to a profile minimum before rebounding in deeper horizons, effectively decoupling the shallow root zone from deeper subsoil moisture and establishing a transitional barrier characteristic of desert–oasis ecotones [37,38,39]. It is noteworthy that the S-shaped vertical moisture distribution was not entirely uniform across all sandy habitats. At Gaotai Heiquan (Site 4) and Linze Sand Farm (Site 6), the vertical moisture profiles exhibited more dampened or modified fluctuations compared to other shifting sand plots. This divergence is primarily attributable to site-specific micro-environmental and edaphic properties. Gaotai Heiquan contains a higher proportion of gravelly sand, which alters macropore continuity and matric suction gradients, smoothing out distinct moisture inflection points. In contrast, Linze Sand Farm exhibits much higher total plant cover (79.01%). Dense shrub root networks and canopy shading redistribute near-surface and subsoil moisture via local hydraulic redistribution and transpiration, which weakens the sharp boundary effect of the DSL [40,41].

Constrained by the DSL, soil water availability and vertical distribution serve as the key abiotic filters regulating plant community assembly, floristic diversity, and synusial structure [18]. In sandy habitats, the 20–300 cm horizon forms the primary distribution zone and physiological absorption depth for desert root systems [42]. Deep-rooted woody trees appear unable to effectively penetrate the compact, desiccated DSL at 60–80 cm depth to access deeper subsoil moisture. This excludes arboreal species entirely from the natural ecotone, so vegetation is limited to shallow-rooted xerophytic shrubs, semi-shrubs, and herbaceous plants [43]. These shallow-rooted synusiae mainly use water from the active 0–60 cm layer, yet subsoil moisture reserves below the DSL provide an important hydrological baseline. This stable subsoil reservoir moderates thermal and moisture gradients in the overlying profile through microscale vapor diffusion, easing severe drought stress and indirectly supporting plant community diversity and cover [12]. These inferences were obtained by fitting SEM to observational cross-sectional data and reflect statistical path associations rather than direct experimental uptake mechanisms. Further validation using stable isotope probing is therefore required [44]. Consequently, the community maintains an austere structural hierarchy dominated by shrub and herbaceous synusiae, with shrubs dominated by *R. songarica* and subdominated by *S. passerina*, while the herbaceous layer is dominated by the annual grass *A. adscensionis* alongside the perennial sedge *C. tristachya* [45,46]. The pronounced dominance of ephemeral and annual plants reflects a specialized evolutionary phenological adaptation under DSL control: annuals rapidly capitalize on transient precipitation pulses within the upper 0–40 cm sand layer during the brief rainy season to complete their reproductive life cycle, whereas perennial shrubs rely on extensive lateral root branches spread throughout the 20–60 cm horizon to exploit moisture stored above the DSL during dry intervals [47]. This edaphic constraint ultimately explains the low species richness, simplified horizontal patterning, and inherent structural fragility of vegetation communities across the regional sand margin [48].

The high correlation coefficients (r ≥ 0.93) among total vegetative coverage, shrub coverage, and herbaceous coverage provide empirical support for the hypothesis of synusial co-development in hyper-arid ecotones [49]. Canopy closure correlated strongly with all coverage metrics (r ≥ 0.93), indicating that shrub canopy density serves as an effective proxy for understory herbaceous establishment by regulating microclimatic illumination, buffering near-surface temperatures, reducing soil evaporation, and fostering fertile-island nutrient patches [50,51,52]. In contrast, the weak negative correlation observed between soil water content and vegetation cover metrics (−0.33 ≤ r ≤ −0.24) suggests that high-coverage vegetation clusters accelerate localized soil moisture consumption through intense transpirational drawdowns, leading to localized soil drying [53]. Furthermore, elevation exhibited moderate positive correlations with total coverage (*r* = 0.49) and soil water content (*r* = 0.41), indicating that topographic elevation along the alluvial fan gradient improves micro-hydrothermal combinations and reduces human disturbance, thereby reinforcing natural vegetation development and profile water retention [54,55]. These microclimatic and structural facilitative effects also explain the broad Shannon–Wiener niche breadths of constructive shrubs (*R. songarica*, ***B_SW_*** = 0.368; *S. passerina*, ***B_SW_*** = 0.316) and the high interspecific niche overlap (>0.86) observed across dominant shrub pairs. This pattern reflects high similarity in spatial environmental distribution rather than interspecific competitive exclusion, which may facilitate species coexistence within this moisture-constrained transition belt [56,57]. Similarly, the pervasive clumped spatial pattern revealed across all dominant populations (*C* = 1.98–89.85, PAI > 1.0) illustrates an adaptive spatial aggregation strategy whereby plants cluster within favorable soil moisture depressions to alleviate evaporative demands and enhance population survivorship [58,59].

The natural DSL acts as an irreplaceable hydrological barrier that suppresses evaporation and preserves subsoil moisture. Ecological restoration policies should avoid blind pursuits of high-density artificial afforestation or uniform greening. Planting water-intensive, deep-rooted trees (such as *P. euphratica*) in sand dune zones disrupts the natural hydrological balance and excessively mines deep subsoil water pools beneath the DSL. This practice can potentially trigger irreversible soil desiccation layer formation, leading to wide-scale plantation degradation [60,61]. Sand stabilization initiatives should retain a baseline proportion of natural bare sandland to foster the spontaneous development and self-maintenance of the DSL, thereby safeguarding groundwater reservoirs and landscape hydrological equilibrium [62]. In artificial vegetation reestablishment programs, priority should be given strictly to indigenous, drought-tolerant, shallow-rooted xerophytic shrubs and grasses (*R. songarica*, *S. passerina*, *A. adscensionis*, and *C. songorica*) whose root architectures match the 0–60 cm soil moisture zone, coupled with passive rainwater harvesting and soil moisture conservation techniques to enhance seedling establishment.

Across the globe, extensive hyper-arid and arid transition zones within terrestrial drylands commonly face severe water limitation and comparable edaphic crusting or dry-sand layer features. Our profile-scale coupling framework links mechanical soil compaction, DSL-mediated vapor–liquid decoupling, and microscale plant synusial assembly. It provides a generalizable process-based framework for exploring plant–soil interactions in other fragile dune interdune ecosystems worldwide. Practically, these results highlight substantial ecological risks from overzealous high-density afforestation using deep-rooted trees in water-limited environments, consistent with the growing global consensus to respect natural hydrological thresholds in dryland restoration [63]. Future international management strategies should pivot toward protecting native edaphic boundaries and deploying shallow-rooted native species to achieve long-term ecological sustainability and climate resilience in global drylands [64]. Because this investigation did not track temporal groundwater table oscillations and interannual precipitation variability, subsequent long-term research should integrate continuous spatiotemporal monitoring of groundwater–soil–vegetation water fluxes to further elucidate eco-hydrological adaptation mechanisms across desert–oasis ecotones.

## 5. Conclusions

In the desert–oasis ecotone of the middle Heihe River Basin, the DSL consistently forms at a depth of 60–80 cm with a thickness of 12–20 cm, exhibiting a distinct moisture trough and the weakest water retention across the 0–120 cm profile. The vertical soil moisture distribution conforms to a distinct S-shaped increase–decrease–increase pattern that is significantly constrained by soil compaction. Acting potentially as an eco-hydrological gatekeeper, the DSL is hypothesized to exert a dual regulatory mechanism that attenuates vertical infiltration to retain scarce rainfall within the shallow 0–60 cm root zone while severing capillary connectivity to suppress subsoil evaporation, thereby maintaining regional water balance. This edaphic stratification acts as an environmental filter that largely restricts deep-rooted trees and confines community assembly to shallow-rooted xerophytic shrubs (*R. songarica*, *S. passerina*) and companion grasses (*A. adscensionis*, *C. tristachya*), which exhibit high interspecific niche overlap (>0.86) and clumped spatial distributions (*C* = 1.98–89.85, PAI > 1.0). Consequently, regional ecological restoration should be guided to prioritize the protection and natural development of the DSL and deploy indigenous, drought-hardy shallow-rooted shrubs and grasses aligned with shallow soil water availability.

## Figures and Tables

**Table 1:** Geographical and ecological characteristics of the six study sites.

Site ID	Location Name	Elevation (m)	Soil Type	Total Cover (%)	Dominant Shrub	Dominant Herb
Site 1	Nantan	1848.9	Silt loam	64.57	*Salsola passerina* *Bunge*	*Carex tristachya* Thunb.
Site 2	Tu’erbatan	1485.9	Sand	35.25	*Reaumuria songarica* (Pall.) Maxim.	*Aristida adscensionis* L.
Site 3	Banqiao	1395.9	Sand	22.31	*R. songarica* (Pall.) Maxim.	*Artemisia scoparia* Waldst. & Kit.
Site 4	Heiquan	1344.8	Gravelly sand	25.00	*R. songarica* (Pall.) Maxim.	*Artemisia xerophytica* Krasch.
Site 5	Nanhua	1426.4	Fine sand	19.67	*R. songarica* (Pall.) Maxim.	*S*. *passerina* Bunge
Site 6	Sand Control Farm	1385.9	Sand	79.01	*R. songarica* (Pall.) Maxim.	*Bassia dasyphylla* (Fisch. et Mey.)

**Table 2:** Soil bulk density across the 0–120 cm depth profiles among the six study sites in the desert–oasis ecotone (g. cm^−3^).

Soil Depth (cm)	Site 1	Site 2	Site 3	Site 4	Site 5	Site 6	Mean ± SD
0–10	1.40	1.69	1.76	1.48	1.72	1.74	1.63 ± 0.15
10–20	1.24	1.59	1.58	1.41	1.66	1.63	1.52 ± 0.16
20–40	1.36	1.52	1.49	1.19	1.57	1.47	1.43 ± 0.14
40–60	1.32	1.62	1.62	1.49	1.48	1.63	1.53 ± 0.12
60–80 (DSL)	1.29	1.49	1.40	1.39	1.38	1.38	1.39 ± 0.06
80–100	1.23	1.43	1.32	1.38	1.38	1.43	1.36 ± 0.08
100–120	1.17	1.41	1.26	1.34	1.31	1.22	1.29 ± 0.09
Mean ± SD	1.29 ± 0.08	1.54 ± 0.10	1.49 ± 0.17	1.38 ± 0.10	1.50 ± 0.16	1.50 ± 0.20	1.45 ± 0.12

**Table 3:** Plant traits and importance values in the desert–oasis ecotone of the middle Heihe River Basin.

No.	Species	Family	Life Form	Importance Value (IV, %)
1	*Reaumuria soongarica* (Pall.) Maxim	Tamaricaceae	Shrub/Semi-shrub	111.17
2	*Salsola passerina* Bunge	Chenopodiaceae	Shrub/Semi-shrub	54.90
3	*Nitraria tangutorum* bobrov	Zygophyllaceae	Shrub/Semi-shrub	24.42
4	*Sympegma regelii* Bunge	Chenopodiaceae	Shrub/Semi-shrub	20.11
5	*Calligonum mongolicum* Turcz.	Polygonaceae	Shrub/Semi-shrub	18.74
6	*Caragana jubata* (Pall.) Poir.	Fabaceae	Shrub/Semi-shrub	18.68
7	*Artemisia frigida* Willd.	Asteraceae	Shrub/Semi-shrub	18.46
8	*Artemisia xerophytica* Krasch.	Asteraceae	Shrub/Semi-shrub	16.58
9	*Asterothamnus centraliasiaticus* Novopokr.	Asteraceae	Shrub/Semi-shrub	8.13
10	*Hedysarum scoparium*	Fabaceae	Shrub/Semi-shrub	5.31
11	*Aristida adscensionis* L.	Poaceae	Herbaceous	48.62
12	*Carex tristachya* Thunb.	Cyperaceae	Herbaceous	23.39
13	*Chloris virgata* Sw.	Poaceae	Herbaceous	18.22
14	*Bassia dasyphylla* (Fisch. et Mey.)	Chenopodiaceae	Herbaceous	13.61
15	*Cleistogenes songorica* (Roshev.) Ohwi	Poaceae	Herbaceous	13.61
16	*Salsola paulsenii* Litv.	Chenopodiaceae	Herbaceous	11.84
17	*Artemisia scoparia* Waldst. & Kit.	Asteraceae	Herbaceous	11.40
18	*Artemisia capillaris* Thunb.	Asteraceae	Herbaceous	10.47
19	*Stipa tianschanica* Roshev.	Poaceae	Herbaceous	10.36
20	*Echinops sphaerocephalus* Trevir.	Asteraceae	Herbaceous	10.02
21	*Salsola ruthenica* Iljin	Chenopodiaceae	Herbaceous	9.21
22	*Eragrostis pilosa* (L.) Beauv. var. pilosa	Poaceae	Herbaceous	8.69
23	*Achnatherum splendens*	Poaceae	Herbaceous	4.82
24	*Limonium aureum* var. maduoense	Plumbaginaceae	Herbaceous	3.77
25	*Lithospermum fimbriatum*	Boraginaceae	Herbaceous	3.19
26	*Tragus mongolorum* Ohwi	Poaceae	Herbaceous	3.18
27	*Lepidium apetalum* Willd.	Brassicaceae	Herbaceous	2.61
28	*Agriophyllum squarrosum*	Chenopodiaceae	Herbaceous	2.02
29	*Cynanchum sibiricum* Willd.	Apocynaceae	Herbaceous	1.99
30	*Astragalus tataricus* Franch.	Fabaceae	Herbaceous	1.81
31	*Corispermum hyssopifolium*	Chenopodiaceae	Herbaceous	1.78
32	*Peganum harmala* L.	Zygophyllaceae	Herbaceous	1.72
33	*Halogeton arachnoideus*	Chenopodiaceae	Herbaceous	1.71
34	*Bothriospermum chinense* Bunge	Boraginaceae	Herbaceous	1.67
35	*Arnebia szechenyi* Kanitz	Boraginaceae	Herbaceous	1.61
36	*Suaeda glauca* (Bunge) Bunge	Chenopodiaceae	Herbaceous	1.59
37	*Zygophyllum fabago* L.	Zygophyllaceae	Herbaceous	1.59

Note: Values in the table represent the Importance Value (IV, %), calculated as the sum of relative density, relative frequency, and relative coverage (totaling up to 300%).

## Data Availability

The original contributions presented in this study are included in the article. Further inquiries can be directed to the corresponding author.

## Supplementary Materials

The following supporting information can be downloaded at https://www.mdpi.com/article/10.3390/biology15211943/s1, Table S1: Vertical distribution of soil particle-size composition across depth intervals; Table S2: Plant community survey attributes and importance values across study sites; Table S3: Summary of plant community alpha diversity indices.

## Abbreviations

The following abbreviations are used in this manuscript:

DSL	Dry-Sand Layer
SEM	Structural Equation Model
RDA	Redundancy Analysis
BD	Bulk Density
SWC	Soil Water Content
IV	Importance Value
OLS	Ordinary Least Squares
HSD	Honest Significant Difference
